# 

**Published:** 1875-06-15

**Authors:** 


### MEDICAL EXAMINER:

A

### Semi-Monthly Journal of Medical Sciences.

EDITED BY

N. S. DAVIS, M. D., and F. H. DAVIS, M. D.

*Subscription Price, Three Dollars per Annum, in advance.*
*Single Numbers, Fifteen Cents.*

CHICAGO.

PUBLISHED AT 65 E. RANDOLPH STREET.

